# Role of ammonia-lyases in the synthesis of the dithiomethylamine ligand during [FeFe]-hydrogenase maturation

**DOI:** 10.1016/j.jbc.2024.107760

**Published:** 2024-09-10

**Authors:** Adrien Pagnier, Batuhan Balci, Eric M. Shepard, Hao Yang, Alex Drena, Gemma L. Holliday, Brian M. Hoffman, William E. Broderick, Joan B. Broderick

**Affiliations:** 1 Department of Chemistry and Biochemistry, Montana State University, Bozeman, Montana, USA; 2 Department of Chemistry, Northwestern University, Evanston, Illinois, USA; 3 Digitisation, Pharmaceutical Science, Biopharmaceuticals R&D, AstraZeneca, Macclesfield, United Kingdom

**Keywords:** [FeFe]-hydrogenase, dithiomethylamine, ammonia lyase, ammonium, glycine cleavage system, hyd operon, HydF, GTPase

## Abstract

The generation of an active [FeFe]-hydrogenase requires the synthesis of a complex metal center, the H-cluster, by three dedicated maturases: the radical *S*-adenosyl-l-methionine (SAM) enzymes HydE and HydG, and the GTPase HydF. A key step of [FeFe]-hydrogenase maturation is the synthesis of the dithiomethylamine (DTMA) bridging ligand, a process recently shown to involve the aminomethyl-lipoyl-H-protein from the glycine cleavage system, whose methylamine group originates from serine and ammonium. Here we use functional assays together with electron paramagnetic resonance and electron-nuclear double resonance spectroscopies to show that serine or aspartate together with their respective ammonia-lyase enzymes can provide the nitrogen for DTMA biosynthesis during *in vitro* [FeFe]-hydrogenase maturation. We also report bioinformatic analysis of the *hyd* operon, revealing a strong association with genes encoding ammonia-lyases, suggesting important biochemical and metabolic connections. Together, our results provide evidence that ammonia-lyases play an important role in [FeFe]-hydrogenase maturation by delivering the ammonium required for dithiomethylamine ligand synthesis.

Hydrogenases are metalloenzymes that catalyze the reversible reduction of protons to H_2_ and are fundamental for microbial energy metabolism (1). [FeFe]-hydrogenases have attracted attention in the field of renewable energy research as a model catalyst for the production and use of hydrogen (2, 3, 4, 5). H_2_ formation is catalyzed in the [FeFe]-hydrogenase active site at a biologically unique hexanuclear iron cofactor, the ‘H-cluster,’ which consists of a four-cysteine ligated [4Fe-4S] cluster ([4Fe-4S]_H_) connected to an organometallic [2Fe] subcluster ([2Fe]_H_) *via* a cysteine residue. The iron atoms of the [2Fe]_H_ are coordinated by carbonyl (CO) and cyanide (CN^–^) ligands, as well as a bridging dithiomethylamine (DTMA) ligand (Fig. 1), and in its oxidized form (denoted H_ox_; [4Fe-4S]^II^ - [Fe]_p_^I^ - [Fe]_d_^II^) gives an electron paramagnetic resonance (EPR) signal with g = [2.103, 2.044, 1.998] (6, 7, 8). Maturation of the [FeFe]-hydrogenase into an active enzyme requires the assembly of the H-cluster in a complex stepwise process that starts with the insertion of the [4Fe-4S]_H_ cluster into the hydrogenase by housekeeping Fe-S cluster biogenesis machinery (9, 10, 11), followed by assembly and insertion of the [2Fe]_H_ subcluster by three dedicated maturation enzymes: HydG, HydE and HydF (12, 13, 14, 15).

**Figure 1:**
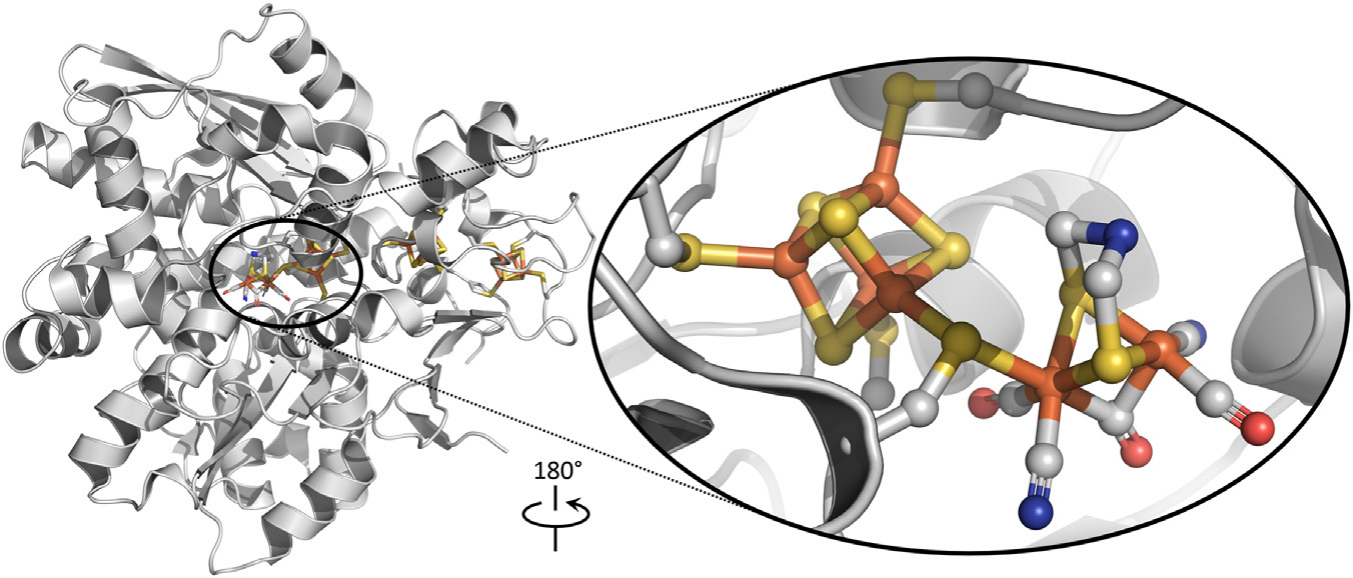
**[FeFe]-hydrogenase from *Clostridium pasteurianum* (3C8Y) and its active site H-cluster (inset)**. *Atom color*, identity: *rust*, iron; *yellow*, sulfur; *blue*, nitrogen; *red*, oxygen; *gray*, carbon.

In the first step of [FeFe]-hydrogenase maturation, the radical *S*-adenosyl-l-methionine (SAM) enzyme HydG lyses tyrosine at the radical SAM (RS) [4Fe-4S] cluster to produce a 4-oxidobenzyl radical and dehydroglycine (16). The dehydroglycine is subsequently cleaved into CO and CN^–^ diatomic ligands which bind to the unique fifth “dangler’’ iron of an auxiliary [5Fe-4S] cluster on HydG, ultimately generating a [Fe^II^(Cys)(CO)_2_(CN)]^–^ synthon (17, 18, 19, 20, 21, 22, 23). Recent work has shown that the synthon is delivered directly from HydG to the second RS enzyme HydE (24). It was shown that HydE binds a synthetic analog of this synthon (synB) and adenosylates it, similar to the adenosylation reaction HydE catalyzes with thiazolidine substrates (25, 26). The HydE-catalyzed synB adenosylation reaction was shown by EPR spectroscopy to result in the reduction of the [Fe^II^(Cys)(CO)_2_(CN)]^–^ to an Fe^I^ synthon species, and HydE was proposed to subsequently dimerize two Fe^I^ synthon units to form the dinuclear [(Fe^I^)_2_(*μ*-SH)_2_(CN)_2_(CO)_4_]^2–^ complex ([2Fe]_E_) (27). The [2Fe]_E_ cluster was then proposed to be transferred to HydF, a multidomain [4Fe-4S] cluster-containing scaffold protein with GTPase activity, prior to the installation of the bridging DTMA ligand (28, 29, 30, 31, 32, 33, 34, 35, 36, 37, 38, 39, 40, 41, 42, 43). Support for this model was provided by recent work demonstrating that the use of a synthetic [Fe_2_(*μ*-SH)_2_(CN)_2_(CO)_4_]^2–^ complex, the presumed product of HydE, allowed the maturation of the [FeFe]-hydrogenase in the absence of HydG and HydE (44).

Since the methodology for *in vitro* maturation of [FeFe]-hydrogenase was first developed in 2008 (45), there has been an absolute requirement for the presence of clarified *Escherichia coli* (*E. coli*) lysate, indicating that the lysate was providing one or more unidentified but essential components for maturation. The crucial lysate component was recently identified as the aminomethyl-lipoyl-H-protein (H_met_) of the glycine cleavage system (GCS, Fig. S1), a discovery that led to the development of a lysate-free defined maturation system involving serine hydroxymethyltransferase (SHMT) and the T-protein of GCS in place of the *E. coli* lysate (Fig. 2) (14). Using this defined system, matured [FeFe]-hydrogenase was produced and characterized by electron-nuclear double resonance (ENDOR) spectroscopy, revealing that serine and ammonium provide the carbon and nitrogen, respectively, of the DTMA ligand of the H-cluster. This result apparently contradicted a prior lysate-based maturation pointing to serine as the source of both the N and C atoms of DTMA but raised the question of whether serine ammonia-lyase in the *E. coli* lysate might liberate NH_3_ from serine in reactions containing lysate (14, 46). A fully-defined semisynthetic maturation of the [FeFe]-hydrogenase using the synthetic [(Fe^I^)_2_(*μ*-SH)_2_(CN)_2_(CO)_4_]^2–^ cluster to bypass HydE and HydG revealed that [FeFe]-hydrogenase maturation could be accomplished with only the HydF maturase and the GCS components (47). It was suggested that H_met_ and the GCS interact with a HydF/[(Fe^I^)_2_(*μ*-SH)_2_(CN)_2_(CO)_4_]^2–^ complex to install the DTMA ligand during maturation (47).

**Figure 2:**
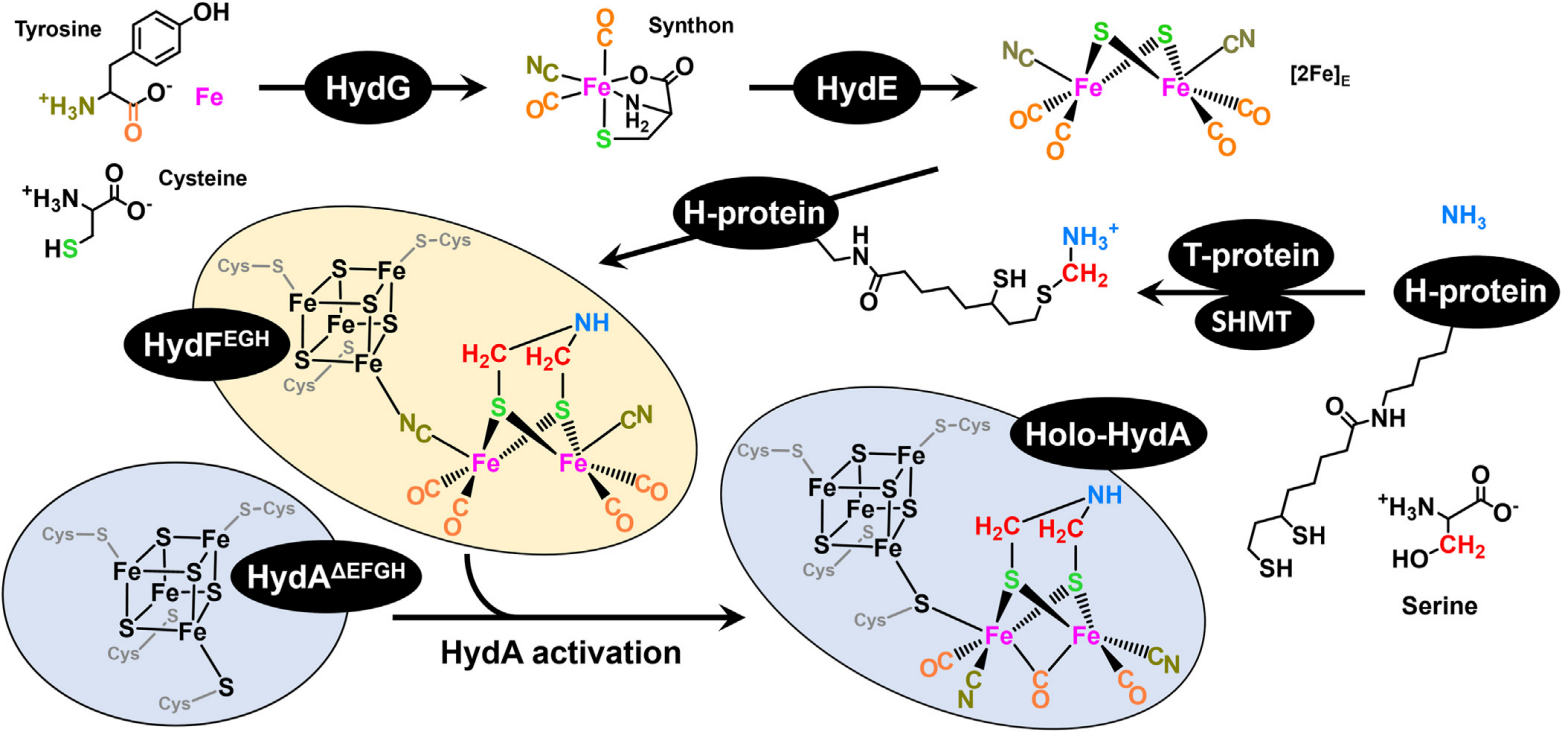
**Schematic depiction of the current model for [FeFe]-hydrogenase (HydA) maturation by the maturases HydG, HydE, and HydF, as well as components of the GCS including H-protein, T-protein, and SHMT, which together convert the HydA**^**ΔEFG**^**containing only the [4Fe-4S]**_**H**_**cluster to the holo-[FeFe]-hydrogenase**.

Here we address the biological origins of the bridgehead nitrogen of the DTMA ligand of the H-cluster during biosynthesis of the H-cluster. We show that in a fully defined maturation system for the [FeFe]-hydrogenase from *Chlamydomonas reinhardtii* (*Cr*HydA), both serine and aspartate can serve as the source of the bridgehead N of DTMA when the corresponding enzymes serine ammonia-lyase and aspartate ammonia-lyase are included in the maturation assay. Further, analysis of the *hyd* operon reveals its close association with genes for ammonia-lyases, suggesting that *in vivo* [FeFe]-hydrogenase maturation is dependent on ammonia-lyases to provide the N atom for DTMA biosynthesis. These results yield important new insights into the biochemical interconnections between [FeFe]-hydrogenase maturation, one-carbon metabolism *via* the GCS, and nitrogen cycling and energy metabolism *via* the ammonia lyase enzymes.

## Results

### Origin of DTMA bridgehead N depends on the presence of lysate

**Figure 3:**
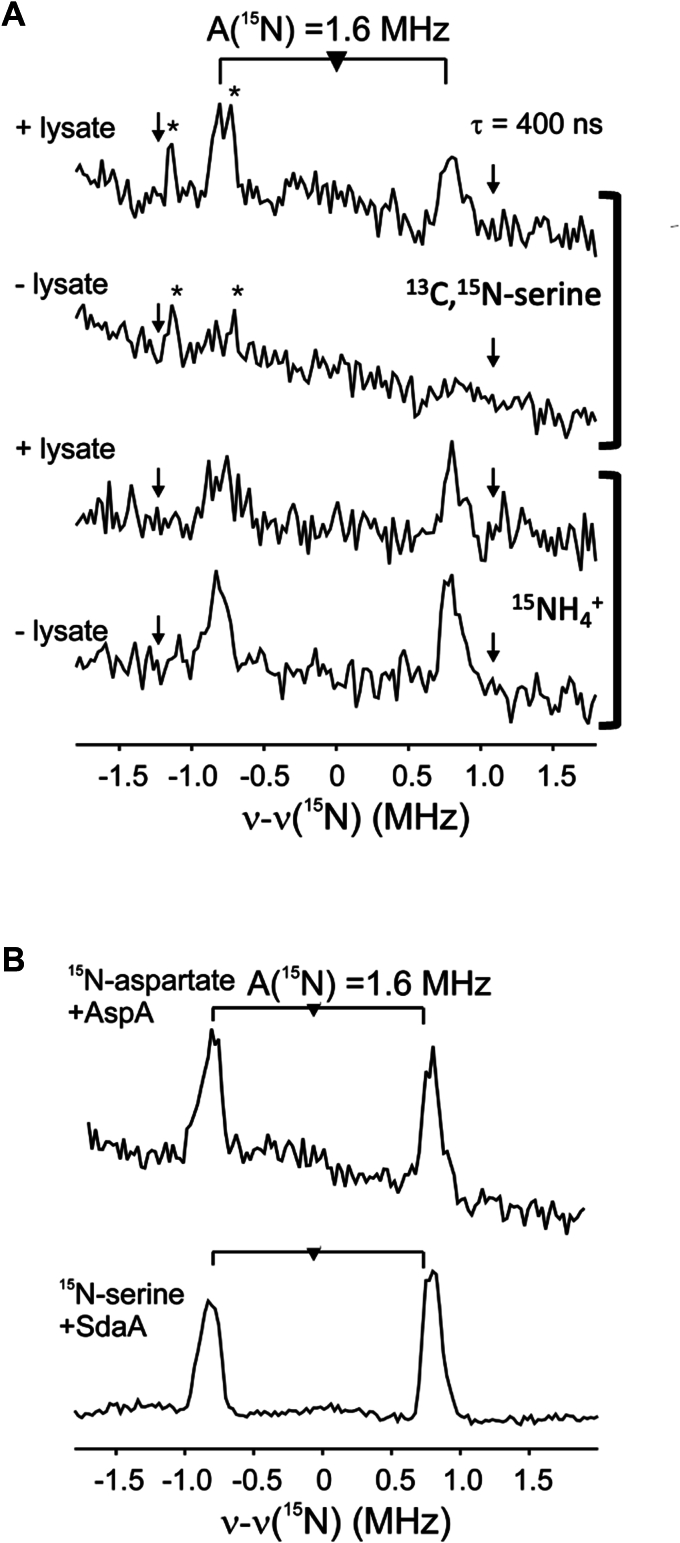
**2K Q-band**^**15**^**N Mims ENDOR spectra centered at the**^**15**^**N Larmor frequency and taken at g = 2.01 of mature HydA**. *A*, Maturation in the presence of ^15^NH_4_^+^ (lower two spectra) results in ^15^N incorporation into DTMA regardless of the presence of lysate, while ^15^N incorporation from ^13^C,^15^N-serine (top two spectra) occurs only in the presence of lysate, indicating a role for an enzyme in the lysate. The bottom spectrum in panel (*A*) is reproduced from reference (14). (∗) represents harmonics of the ^13^C ENDOR response (not shown) from ^13^C incorporated in the DTMA from the doubly labeled serine; (↓) represents Mims ‘holes’ (see Experimental Procedures). *B*, maturation in the presence of ^15^N-aspartate and AspA, or ^15^N-serine and SdaA, results in the incorporation of ^15^N into DTMA, as revealed by ^15^N ENDOR spectra.

[FeFe]-hydrogenase defined *in vitro* maturation reactions were carried out under anaerobic conditions in the presence of *Cr*HydA, the Hyd maturases HydE, HydF, HydG, the GCS proteins H-protein and T-protein, and SHMT as the source of carbon to load methylene-THF on the T-protein. Small molecule components included SAM, tyrosine, cysteine, iron, PLP, serine, NH_4_^+^, GTP, MgCl_2_, DTT, and dithionite (see Methods and SI). The maturation reactions were carried out in the presence or in the absence of lysate, and with ^15^N-serine (which as noted below was also ^13^C labeled) or ^15^NH_4_Cl in place of the unlabeled component. The matured *Cr*HydA was purified from the maturation mixture, oxidized by thionin to the H_ox_ state, and analyzed by EPR (Fig. S2) and ENDOR (Fig. 3) spectroscopies to evaluate the incorporation of the isotopic label into the DTMA of the H-cluster as previously described (14).

*Cr*HydA matured in the presence of ^15^NH_4_Cl shows a ^15^N ENDOR response from the labeled DTMA regardless of whether *E. coli* lysate was present during maturation (Fig. 3*A*, bottom), indicating that nitrogen from NH_4_^+^ is incorporated into DTMA in this defined system. The 1.6 MHz ^15^N coupling observed for the DTMA nitrogen is comparable to that reported previously (14). However, the results shown in Fig. 3*A*, bottom, reveal a decreased intensity of the ^15^N doublet in the ^15^N ENDOR spectra of *Cr*HydA matured with ^15^NH_4_Cl in the presence of lysate, indicating that other sources of unlabeled nitrogen are diluting the labeled nitrogen pool during incorporation into DTMA. *Cr*HydA maturation reactions carried out in the presence of ^13^C,^15^N-serine show ^15^N incorporation into DTMA of the H-cluster, as indicated by the ^15^N doublet with a 1.6 MHz coupling, only when lysate was added to the defined maturation (Fig. 3*A*, top); this suggests that ^15^N can be liberated from ^15^N-serine for incorporation into DTMA by enzymes found in the cell lysate. The ^13^C from ^13^C,^15^N-serine was previously shown to be incorporated into DTMA *via* ^13^C-ENDOR spectroscopy (14). Together, these results point to a potentially important role for *E. coli* ammonia-lyases in providing the nitrogen for DTMA biosynthesis during *in vivo* [FeFe]-hydrogenase maturation during heterologous expression with the Hyd maturases, or when *E. coli* lysate is added to *in vitro* maturation reactions.

### Hyd operon and amino acid ammonia-lyases

**Figure 4:**
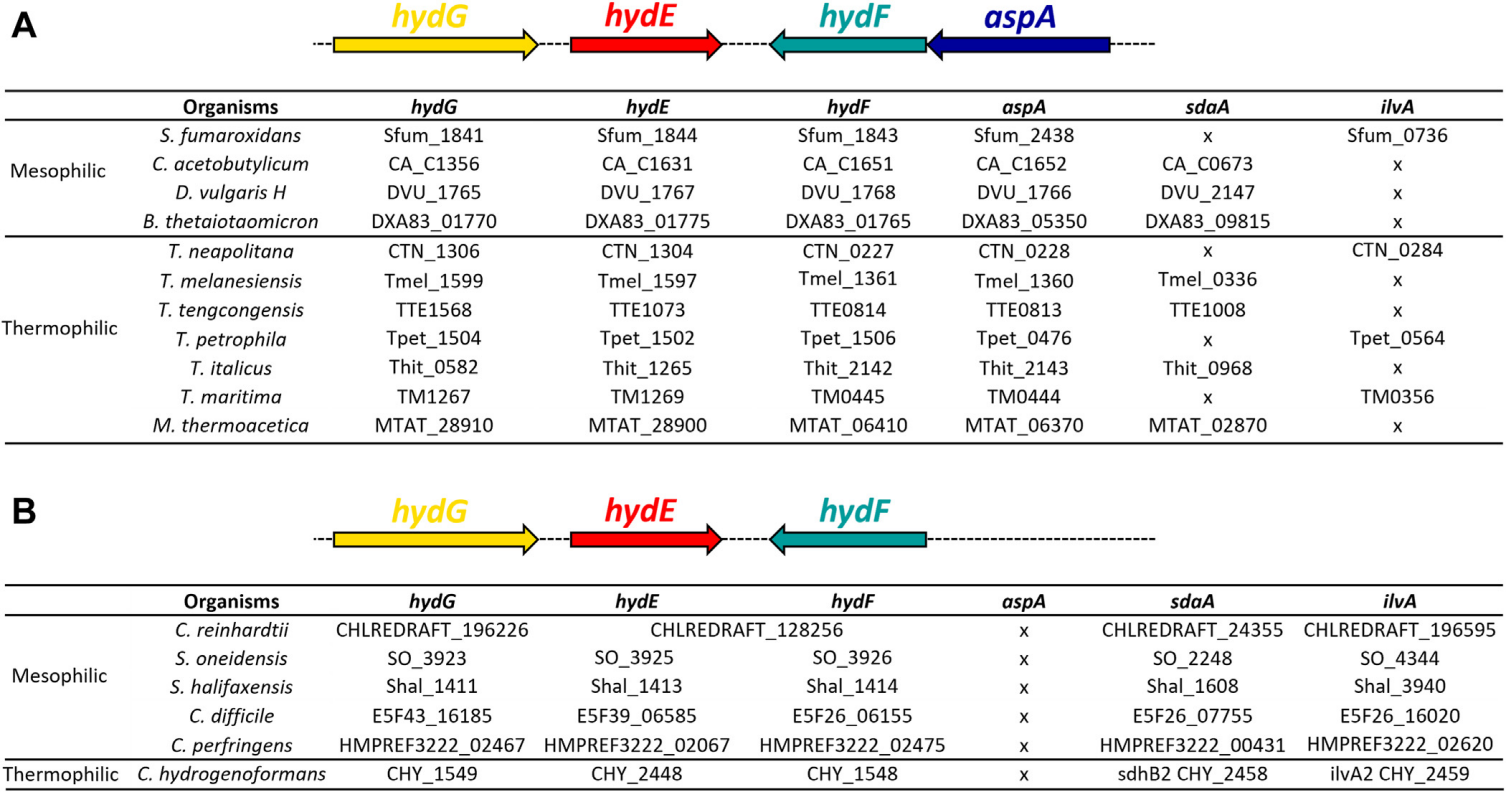
**Select [FeFe]-hydrogenase operons**. *A*, organisms containing the *aspA* gene. *B*, organisms without the *aspA* gene. *aspA*: aspartate ammonia-lyase gene, *sdaA*: serine ammonia-lyase gene, *ilvA*: threonine ammonia-lyase gene. An ‘x’ in the table indicates the corresponding gene is not found.

Analysis of the *hyd* operon from a range of mesophilic and thermophilic organisms reveals that it is closely associated with genes encoding amino acid ammonia-lyases (Fig. 4). Of particular note is the gene *aspA*, coding for aspartate ammonia-lyase (AspA), suggesting that this enzyme could be an important source of ammonia for *in vivo* DTMA synthesis (48). Organisms we identified as having the *aspA* gene associated with the *hyd* operon also invariably have a second ammonia-lyase gene, either *sdaA* encoding serine ammonia-lyase or *ilvA* encoding threonine ammonia-lyase, thus presumably providing an alternate source of ammonia for DTMA biosynthesis. Interestingly, some of the [FeFe]-hydrogenase-producing organisms we examined do not have the *aspA* gene associated with the *hyd* operon; these organisms are found to instead have both the *sdaA* and the *ilvA* genes, thus retaining two potential sources of nitrogen for DTMA biosynthesis in all cases (Fig. 4).

### Bioinformatic analysis of the hyd and amino acid ammonia-lyase genes

**Figure 5:**
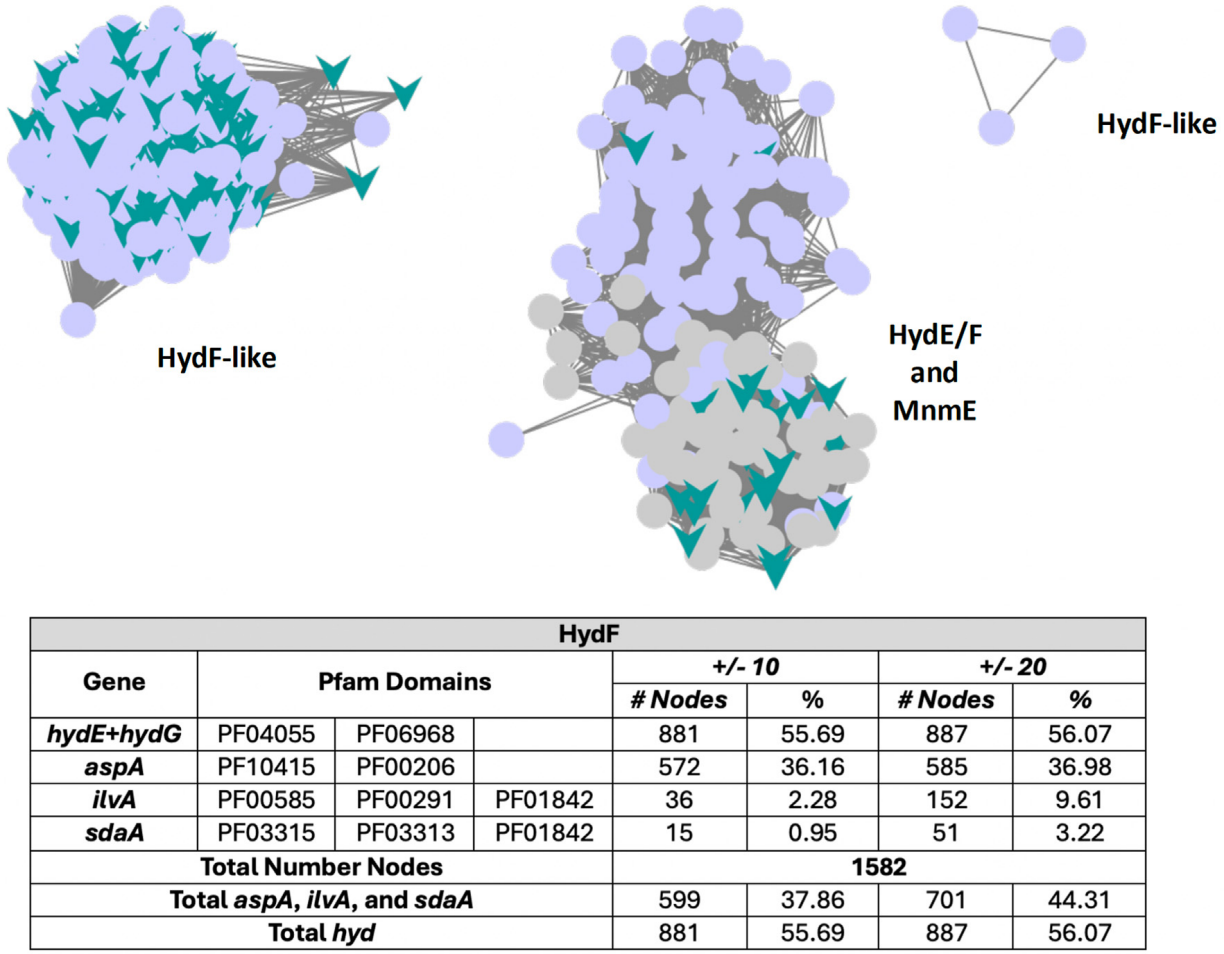
**Genomic context analysis for HydF. Top, sequence similarity network showing the HydF protein set**. The proteins with at least one of the Pfam domains of interest are shown as teal “V’s” and those proteins that have at least one of the hyd gene Pfam domains, but no other Pfam domain of interest are shown as pale purple circles. Each cluster is named with the predominant annotations of proteins in that cluster. Figure created using Cytoscape and a perfuse force-directed layout algorithm. Bottom, a table showing the genes of interest and their associated Pfam domain, the number of nodes for which at least one of those Pfam domains was identified as a genomic neighbor within the ± 10 and ± 20 ORF of *hydF*, and the percentage of the total number of nodes represented.

In order to analyze more broadly the relationships between the *hyd* genes and those for the amino acid ammonia-lyases illustrated in Fig. 4, we constructed sequence similarity networks (SSNs) and genomic context networks (GCNs) as described in the Experimental Procedures. The SSN and GCN for *hydF* (Fig. 5) and *hydE* (Fig. S3) reveal a significant correlation with ammonia lyase genes in the ± 20 open reading frame (ORF) genome neighborhood, as discussed below.

A striking association with ammonia-lyase genes is evident from the bioinformatic analysis of *hydF* genes. For HydF, the number of proteins in the SSN that have at least one of the other *hyd* genes in the ± 20 ORF vicinity of *hydF* is 887 out of 1582 (roughly 56%); this is less than 100% since using BLAST to expand on the protein space will inevitably pull back pseudo-HydF proteins, proteins that look very similar to HydF but do not have the HydF function; we assume that the authentic *hydF* genes are the 887 with adjacent *hyd* maturase genes. The total number of nodes with at least one of the *aspA*, *ilvA*, or *sdaA* genes (as determined by the Pfam domain membership) is 701, or 79% of the authentic *hydF* genes, indicating a strong association that supports the hypothesis that these ammonia-lyases play an important role in providing the ammonia needed for DTMA biosynthesis during *in vivo* [FeFe]-hydrogenase maturation. It is interesting to note that *aspA* in particular is much more likely to be close to *hydF*, *i.e.* within ± 10 ORFs, than either *ilvA* or *sdaA*. The correlation of *aspA*, *ilvA*, or *sdaA* genes with *hydE* is lower (SI), consistent with HydF (and not HydE) being the enzyme involved in DTMA synthesis which requires ammonia as a source of N of DTMA.

### Ammonia-lyases support [FeFe]-hydrogenase maturation in the absence of exogenous NH_4_^+^

Given the prominence of *aspA* near the *hyd* operon in most organisms that have an [FeFe]-hydrogenase, we tested the ability of aspartate ammonia-lyase to provide ammonium during HydA maturation. Aspartate ammonia-lyase is a divalent metal activated enzyme that at alkaline pH catalyzes the reversible reaction of aspartate cleavage into fumarate and ammonium (49, 50). *E. coli* AspA was overexpressed and purified (Figs. S4 and S5), and then included in a lysate-free maturation reaction containing ^15^N-aspartate; the matured HydA was analyzed by ENDOR spectroscopy after repurification from the reaction mixture (Fig. 3*B*). The results reveal a ^15^N doublet with hyperfine coupling of 1.6 MHz, consistent with the incorporation of ^15^N from ^15^N-aspartate into the DTMA of the H-cluster.

Serine ammonia-lyase (SdaA) is a [4Fe-4S] cluster-dependent enzyme present in *E. coli* lysate that catalyzes the liberation of NH_4_^+^ from serine (51). We and others have proposed that SdaA could be responsible for the observed incorporation of nitrogen from serine into DTMA in maturation reactions that include lysate (14, 46). To test this hypothesis, we purified and reconstituted *E. coli* SdaA (see SI Methods and Figs. S4, S6, and S7) and carried out a maturation reaction in which SdaA and ^15^N-serine were added to lysate-free maturation reactions that did not contain NH_4_Cl. ENDOR spectroscopy of the holo-*Cr*HydA purified after maturation reveals the incorporation of ^15^N from serine into DTMA as evidenced by a ^15^N doublet with hyperfine coupling of 1.6 MHz, demonstrating that SdaA can provide the nitrogen from serine for use in DTMA biosynthesis (Fig. 3*B*).

Together, these results show that ammonia-lyases can liberate ammonium from multiple sources for use during HydA maturation, and provide a biochemical connection that reflects the proximity of ammonia-lyase genes to the *hyd* gene cluster. Further, our findings clarify why previous results have shown that for *in vitro* maturations that included *E. coli* lysate, serine is a source of the DTMA nitrogen: because serine ammonia lyase is a component of the lysate (46).

**Figure 6:**
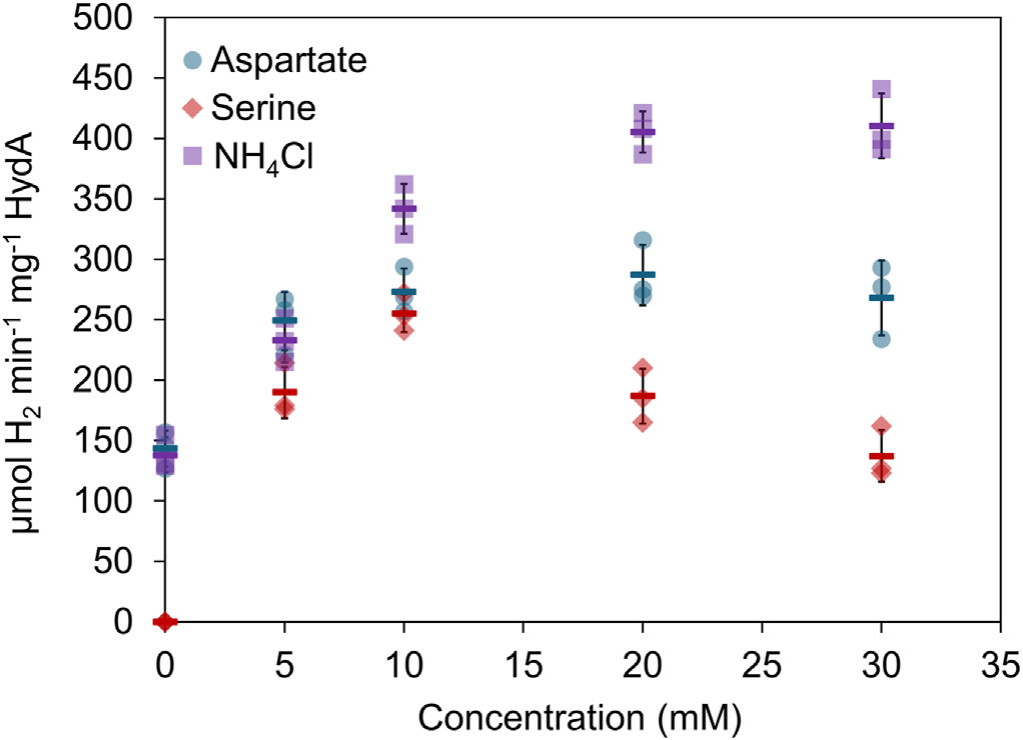
**Specific activities of HydA matured under defined conditions using the maturases HydE, HydF, and HydG in the presence of different sources of ammonium**. The source of ammonium is indicated by the color (*purple*, ammonium chloride; *blue*, aspartate, and AspA; *red*, serine, and SdaA). Assays were conducted in triplicate; individual results are shown as transparent colored shapes with the mean as a colored line and the error bars are shown in *black*. Assay conditions are as described in the supplemental methods.

To further probe the involvement of ammonia-lyases as the source of ammonium during defined *in vitro* HydA maturation reactions, we examined the dependence of HydA maturation on serine and aspartate concentrations, in the presence of their respective ammonia-lyases. The results reveal a dependence of HydA maturation on the concentrations of aspartate and serine that is similar to the ammonium concentration dependence previously reported (Fig. 6). The maturation observed with 0 mM added nitrogen source in the case of the NH_4_^+^ (Fig. 6 – purple) and aspartate/AspA (Fig. 6 – blue) reactions could result from co-purification of NH_4_^+^ with T-protein, due to the well-defined NH_4_^+^ binding site in the T-protein (52). Alternatively, the decomposition of cysteine in the reaction mixture to produce pyruvate, sulfide, and ammonia is a known reaction in the presence of transition metals like iron (53, 54), and promoted *in vitro* by the addition of PLP (55). Because cysteine, iron, and PLP are all included in these defined *in vitro* HydA maturation reactions, there is likely always a background level of NH_4_^+^ available in solution for DTMA biosynthesis.

No maturation is detected at 0 mM serine for the SdaA reactions (Fig. 6 – red), which is consistent with the double role of serine as a source of the C (*via* SHMT and T-protein, Fig. 2) and N (*via* SdaA) of DTMA under these conditions. The presence of DTMA of ^13^C from the doubly-labeled serine has been previously reported (14), and is indicated here by the appearance of the ^13^C harmonic peaks in Fig. 3*A*. Neither the aspartate/AspA nor the serine/SdaA reactions achieve the same levels of maturation as the NH_4_^+^ reactions at concentrations above 10 mM; this likely in part reflects the limitations of the turnover rates of the ammonia-lyases under the conditions of the maturation reactions. In addition, the pyruvate product of serine turnover by SdaA inhibits [FeFe]-hydrogenase maturation in a concentration-dependent manner (Fig. S8), thus explaining the decreased [FeFe]-hydrogenase activity observed at higher concentrations of serine (Fig. 6, red).

## Discussion

**Figure 7:**
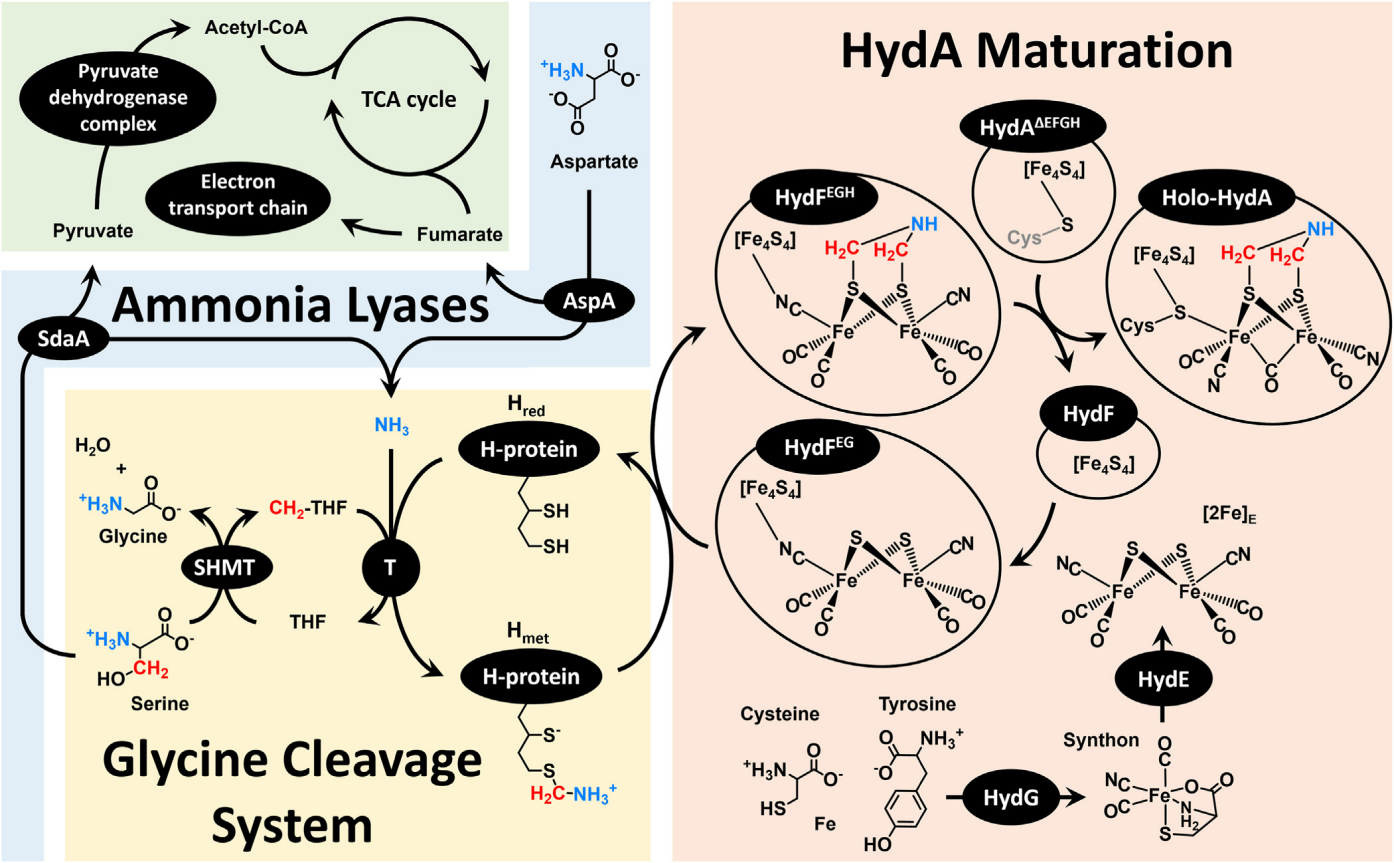
**Role of ammonia-lyases in HydA maturation**.

Our results clarify the source of the bridgehead N of DTMA, by showing that serine can serve as an indirect source of nitrogen *via* the production of ammonium when serine ammonia-lyase is provided by cell lysate or added in its purified form (46). We further showed that another ammonia-lyase, AspA, also can provide the bridgehead nitrogen of DTMA by releasing ammonium from aspartate. The relatively high degree of conservation of *aspA* in the *hyd* operon neighborhood (Figs. 4 and 5) supports an important role for AspA and aspartate as a source of ammonium during HydA maturation *in vivo*. Of note, organisms that have the *aspA* gene in the *hyd* operon also have either serine or threonine ammonia-lyase enzymes, presumably as a supporting ammonium source that may speak to metabolic fluxes during *in vivo* synthesis of the H-cluster (Fig. 4). Interestingly, many organisms that do not have the *aspA* gene in the *hyd* operon have both *sdaA* and *ilvA* genes, and could use the resulting ammonia-lyases (serine and threonine respectively) as sources of ammonium for the GCS. Serine ammonia-lyase is closely linked to the GCS and one-carbon metabolism, strongly suggesting that SdaA is a natural source of ammonium during *in vivo* [FeFe]-hydrogenase maturation (Fig. 7) (56).

The relatively high degree of conservation of *aspA* in the *hyd* operon raises a question: why is it conserved over the other ammonia-lyases? Both SdaA and AspA can provide ammonia for the GCS and feed the TCA cycle *via* their by-products, pyruvate and fumarate respectively, but the fumarate produced by AspA can also be used as an electron acceptor for anaerobic respiration (Fig. 7). Given that most organisms producing the [FeFe]-hydrogenase are anaerobes, there would be an evolutionary advantage to AspA/aspartate over SdaA/serine as the source of nitrogen for DTMA biosynthesis. Consistent with this idea, organisms harboring the *hyd* operon but no *aspA* use other electron acceptors than fumarate: *Shewanella oneidensis* uses Fe^III^, Mn^IV^, and U^VI^ as terminal electron acceptors, and others use sulfate, sulfur, nitrate, or nitrite (57, 58, 59, 60, 61, 62, 63, 64). In addition, it is interesting to note that the *aspA* gene is almost always in the immediate proximity of the *hydF* gene (Figs. 4 and 5) that encodes for the HydF maturase implicated in DTMA synthesis (14, 44). Moreover, HydF was shown to belong to a subclass of GTPase enzymes whose activity is gated by monovalent cations; intriguingly, ammonia was shown to stimulate GTP hydrolysis by HydF (41), further supporting the connection between HydF and AspA.

The recognition of the interconnection between the HydA biosynthetic pathway and ammonia-lyases *via* the GCS confirms the key physiological roles played by the GCS as an important part of the one-carbon metabolic pathway in microorganisms (65, 66). Indeed, in addition to its central role in ammonium trafficking between the ammonia-lyases and HydA maturation pathway, the GCS is also coupled to SAM synthesis which is required for the catalytic activity of the maturases HydE and HydG: the GCS is coupled through its main intermediates tetrahydrofolate (THF) and methylene-THF to the tetrahydrofolate cycle and the methionine cycle. The methylene-THF can be further reduced to 5-CH_3_-THF, which is used in the methionine cycle to generate SAM (67).

In the GCS, the methylamine group of H_met_ comes from glycine if the system turns towards glycine cleavage, or serine and ammonium if the system turns toward glycine synthesis (Fig. S1). During *in vitro* HydA maturation, the use of reducing agents such as DTT and NaDT prevents the system from operating in the direction of glycine cleavage *via* the P-protein, since the substrate of the latter enzyme is the oxidized form of the lipoyl-H-protein. The use of strong reducing agents during *in vitro* HydA maturation could be one of the contributing factors as to why serine and ammonia have been reported to be the source of the methylamine group of H_met_ *via* SHMT and T-protein (14). Clearly, reducing conditions are required for the enzymatic activity of HydE and HydG, but the downstream redox flux is unclear for HydF, DTMA biosynthesis, and cluster translocation to HydA. This work begins to clarify some of these points. The presence of *aspA* in the *hyd* operon and its ability to support maturation reinforces the idea that H_met_ produced *in vivo* from serine and ammonia *via* the glycine synthase reactions plays a key role in hydrogenase maturation. It is interesting to note that in some anaerobic bacteria like *Clostridium acidiurici*, *Clostridium cylindrosporum*, or *Clostridium purinolyticum*, the GCS runs mostly in the direction of glycine synthesis as invoked here during hydrogenase maturation (66, 68, 69, 70).

Taken together, our results show that ammonia-lyases are the major source of ammonia required for HydA maturation *via* the GCS and shed light on the central role played by the GCS in HydA maturation. The GCS is coupled to the tetrahydrofolate cycle and central carbon metabolism *via* the glycine-serine-interconversion and the ammonia-lyases to provide the methylene-THF and the ammonium required to synthesize the CNC backbone of DTMA of the H-cluster. The results herein reveal the physiological processes that are tied to [FeFe]-hydrogenase maturation, and further link fundamental metabolic pathways to the chemistry associated with DTMA biosynthesis. Our results connect the picture of HydA maturation to the metabolic pathways of [FeFe]-hydrogenase-containing organisms and significantly increase our understanding of bacterial metabolism during the HydA maturation process.

## Experimental procedures

### Expression, purification, and reconstitution of HisTag maturase enzymes

The expression, isolation, and reconstitution of His-tagged *Clostridium acetobutylicum* (*C.a*.), *Thermotoga maritima* (*T.m*.), and *C. reinhardtii* (*C.r.*) maturases followed previously published protocols: *Tm*HydE (48), *Ca*HydF (37), *Ca*HydG (23) and *Cr*HydA (14). Details are provided in the following paragraphs.

BL21(DE3)RIL cells were transformed with pET21b-*T.m.hydE*. Pre-cultures were started with a single colony selected from the transformation plate in LB media containing 50 μg/ml ampicillin and incubated at 37 °C with 180 rpm shaking overnight. The next day, 10 ml seed culture was used to inoculate 1.5 L phosphate-buffered TB media with 50 μg mL^−1^ ampicillin and incubated at 37 °C 200 rpm shaking. When OD_600_ was 0.5 to 0.7, overexpression was induced with 1 mM IPTG. At the time of induction, ∼0.15 mM ferric ammonium citrate was added into each culture flask and incubated at 37 °C 200 rpm shaking. After 30 min, 0.316 mM L-cysteine was added into each flask and kept incubating at 37 °C for 4 h. After that, a second aliquot of ferric ammonium citrate was added to make ∼0.3 mM final in each flask and incubated at 18 °C 200 rpm shaking overnight. The next day the culture was harvested *via* centrifugation, and the resulting cell pellet was frozen in liquid nitrogen and stored at −80 °C until purification. Cells were lysed in 100 ml 50 mM Tris-HCl pH 8.0, 250 mM KCl buffer containing 8 mg lysozyme, ∼0.1 mg DNase, 200 mg MgCl_2_, 1000 mg Triton X-100, and two tablets of Pierce protease inhibitor tablets for 30 min, homogenized with 18G needle and syringe, at ambient temperature in a Coy chamber followed by centrifuging for 1 h at 4 °C. Clarified lysate was loaded to the Ni-NTA column and equilibrated with 50 mM Tris-HCl pH 8.0, 250 mM KCl, and 10 mM imidazole. Step gradient was applied with increasing imidazole concentration in buffer (50 mM, 150 mM, 225 mM, and 500 mM). The majority of the protein was eluted with 50 mM Tris-HCl pH 8.0, 250 mM KCl, and 150 mM imidazole. The elution fraction was desalted with a desalting column packed with G-25 resin and concentrated with Amicon 30 kDa MWCO spin filters. The final stock was frozen in liquid nitrogen and stored at −80 °C until reconstitution. HydE was reconstituted by incubating the protein with FeCl_3_ and Na_2_S·9H_2_O in 50 mM Tris-HCl pH 8.0, 250 mM KCl, and 5 mM DTT buffer as previously described (48).

Overnight seed culture was started with BL21(DE3) cells transformed with pRSF-*C.a.hydF* construct in LB media containing 50 μg mL^−1^ kanamycin and incubated at 37 °C with shaking overnight. The next day, phosphate-buffered LB media (a total of 9 L distributed equally into 6 2.8 L Fernbach flasks) was supplemented with 5 g L^−1^ D-glucose and 50 μg mL^−1^ kanamycin. After inoculation from the seed culture, flasks were incubated at 37 °C with 230 rpm shaking. When OD_600_ was 0.9 to 1.2, culture flasks were placed into an ice-water bath for 30 min. After cold-shock, cells were induced with 1 mM IPTG, supplemented with ferrous ammonium sulfate, and incubated at 37 °C with 230 rpm shaking for 2.5 h. Then, a second aliquot of FAS was added into each culture flask (∼0.3 mM final FAS concentration in each flask) and sparged with nitrogen gas at 4 °C overnight. The next day cells were harvested by centrifugation at 6000 rpm, 4 °C, and the resulting pellet was frozen in liquid nitrogen. 45 g frozen cell paste was lysed in 90 ml of 50 mM HEPES pH 7.5, 300 mM KCl buffer with 8 mg lysozyme, ∼0.1 mg DNase, 900 mg Triton X-100, 1 mM PMSF, and 180 mg MgCl_2_. Lysate was incubated for ∼45 min and clarified by centrifuging. Clarified lysate supernatant was loaded to the Ni-NTA column and a step gradient was applied with increasing imidazole concentration in 50 mM HEPES pH 7.5, 300 mM KCl buffer (25 mM, 50 mM, 100 mM, and 225 mM). The target protein was eluted with 50 mM HEPES pH 7.5, 300 mM KCl, 225 mM imidazole, and desalted with a desalting column packed with G-25 resin and concentrated with an Amicon 30 kDa MWCO spin filter. The final desalted protein stock was frozen in liquid nitrogen and stored at −80 °C until reconstitution. HydF was reconstituted by following the previously published protocol (34) by incubating with FeCl_3_ and Na_2_S·9H_2_O in 50 mM HEPES pH 7.5, 300 mM KCl, and 5 mM DTT buffer. The final reconstituted and desalted protein stock was concentrated and stored at −80 °C until further use.

Rosetta(DE3)pLysS cells containing pCDF-*C.a.hydG* construct were used to start a pre-culture in LB media containing 25 μg mL^−1^ chloramphenicol and 100 μg mL^−1^ streptomycin. The culture was incubated at 37 °C with shaking overnight. The next day, after diluting the seed culture in a total of 6 L phosphate buffered TB media with antibiotics, culture flasks were incubated at 37 °C with 180 rpm shaking. Cells were induced with 1 mM IPTG at OD_600_ 0.8 to 1.0 and supplemented with ferrous ammonium sulfate and L-cysteine and incubated at 25 °C with 180 rpm shaking overnight. After harvesting cells by centrifugation, wet cell paste was frozen in liquid nitrogen. 40 g frozen cell pellet was lysed in 80 ml of 50 mM Tris-HCl pH 8.0, 250 mM KCl, 5% glycerol containing 10 to 16 mg lysozyme, ∼0.1 mg DNase, 1 mM PMSF, 92 mg MgCl_2_, 800 mg Triton X-100. Lysate was clarified by centrifugation as described above for the other proteins and the clarified lysate supernatant was loaded to the Ni-NTA column. A step gradient was applied by increasing the concentration of imidazole in the buffer. Fractions from 100 to 150 mM imidazole wash were pooled and desalted *via* a desalting column packed with G-25 resin. Desalted protein was concentrated *via* Amicon 50 kDa MWCO spin filters and frozen in liquid nitrogen, stored at −80 °C until reconstitution. HydG was reconstituted as described previously (23) incubating with FeCl_3_ and Na_2_S·9H_2_O in 50 mM Tris-HCl pH 8.0, 250 mM KCl, 5% glycerol, 5 mM DTT buffer. Reconstituted protein stock was desalted, concentrated with spin filters, and stored at −80 °C until use.

### Expression, purification, and reconstitution of StrepTag CrHydA

The preparation of truncated *Cr*HydA (residues 2–56 removed) with a C-terminal StrepTag was performed as described previously (71) with minor modifications (14) as follows. Chemically competent *E. coli* BL21(DE3) cells were transformed with pETDuet-1-*C.r.hydA1* construct and used to start pre-cultures in LB media with 50 μg mL^−1^ carbenicillin. The culture was incubated at 37 °C with shaking overnight. The next day, 50 ml from pre-culture was inoculated into four Fernbach flasks containing phosphate-buffered 1.5 L TB media (a total of 6 L) supplemented 50 μg mL^−1^ carbenicillin. Culture flasks were incubated at 37 °C with 180 rpm shaking. When OD_600_ reached ∼1.1, sterile-filtered IPTG (1 mM final) and ferrous ammonium sulfate (0.5 mM final) were added into each flask and incubated at 37 °C. After 1 h of incubation 0.5 mM L-cysteine was added and kept incubating for 2 h more. After a total of 3 h at 37 °C, the temperature was decreased to 18 °C for overnight incubation with 180 rpm shaking. Cells were pelleted by centrifugation at 7000 rpm at 4 °C. The resulting wet cell paste was frozen in liquid nitrogen and stored at −80 °C. Cells were lysed in 80 ml of 50 mM HEPES pH 8.0, 150 mM KCl buffer containing 8 mg Lysozyme, ∼0.1 mg DNase, 800 mg Triton X-100, 160 mg MgCl_2_, 1 mM PMSF and incubated at ambient temperature for 30 min with continuous stirring. Lysate was clarified by centrifuging at 18,000 rpm for 1 h. Clarified lysate was loaded to 10 ml StrepTactin-XT column equilibrated with 50 mM HEPES pH 8.0, 150 mM KCl buffer. After all the lysate was loaded, the column was washed with the equilibration buffer and the protein was eluted with 50 mM HEPES pH 8.0, 150 mM KCl, 50 mM D-biotin. Eluent was concentrated with Amicon 30 kDa MWCO spin filters and desalted *via* PD-10 column to remove biotin. After desalting, protein stock was concentrated with spin filters, frozen in liquid nitrogen, and stored at −80 °C. HydA was reconstituted by following a previously published protocol (23) using FeCl_3_ as the iron source and Na_2_S·9H_2_O as the sulfur source in 50 mM HEPES pH 8.0, 150 mM KCl, and 5 mM DTT. The final protein stock was desalted *via* a desalting column concentrated with spin filters and stored at −80 °C.

### Expression and purification of HisTag E. coli aminomethyltransferase (T-protein)

The preparation of T-protein with a C-terminal HisTag was performed as described previously (14). Chemically competent *E. coli* cells were transformed with pET23a plasmid containing the *E. coli* T-protein gene. 45 ml seed culture was diluted in Fernbach flasks containing 1.5 L of phosphate-buffered TB media with 100 μg mL^−1^ ampicillin (total culture was 6 L). Culture flasks was incubated at 37 °C with 180 rpm shaking. When the optical cell density, OD_600_, reached 1.1 to 1.9, cells were induced with 1 mM IPTG and incubated at 16 °C overnight with 200 rpm shaking. The next day cells were harvested, frozen in liquid nitrogen, and stored at −80 °C until lysis and purification. 50 g of cell paste was lysed in 100 ml of 50 mM HEPES pH 7.5, 250 mM KCl (Buffer A) containing 8 mg lysozyme, 1000 mg Triton X-100, 1 mM PMSF, 200 mg MgCl_2_ and 0.1 mg DNase. Lysate was incubated at ambient temperature for ∼1 h and clarified by centrifuging at 17,000 rpm for 1 h. Clarified lysate was loaded to a 20 ml Ni-NTA column equilibrated with 50 mM HEPES pH 7.5, 250 mM KCl, and 10 mM imidazole. A step gradient was applied for the purification of his-tagged T-protein by increasing the concentration of imidazole in Buffer A: 10 mM, 50 mM, 100 mM, and 500 mM imidazole. Elution fractions from 50 mM and 100 mM imidazole were combined, concentrated with Amicon Ultra-15 spin filters, and desalted *via* HiPrep 26/10 column equilibrated with Buffer A. Desalted protein was aliquoted, frozen with liquid nitrogen, and stored at −80 °C. H-protein co-purifies with the T-protein and thus H-protein is not added separately to the maturation reactions (14).

### Expression and purification of HisTag E. coli serine hydroxymethyltransferase (SHMT)

The preparation of SHMT with an N-terminal HisTag was performed as described previously (14). Chemically competent BL21(DE3) cells were transformed with pET14b containing *E. coli* SHMT gene. After inoculation from seed culture to Fernbach flasks containing 1.5 L phosphate buffered TB media supplemented with 50 μg mL^−1^ ampicillin, culture flasks were incubated at 37 °C with 180 rpm shaking. Once the cell density, OD_600_, reached ∼1.0, cells were induced with 1 mM IPTG and incubated at 18 °C overnight with 180 rpm shaking. The lysis and purification followed the method described above with the following minor changes. Clarified lysate was loaded to a 20 ml Ni-NTA column equilibrated with 50 mM HEPES pH 7.5, and 250 mM KCl. A step gradient was applied by increasing the imidazole concentration in Buffer A (10 mM, 50 mM, 150 mM, and 500 mM). The target protein was eluted with Buffer A containing 150 mM imidazole. The yellow elution fraction was desalted with a G-25 resin-packed column and concentrated with Amicon spin filters. The final protein stock was aliquoted, frozen with liquid nitrogen, and stored at −80 °C.

### Expression, purification, and reconstitution of E. coli serine deaminase (SdaA)

Pre-cultures of BL21(DE3) cells containing the *sdaA* gene in pET28a plasmid were started in LB media supplemented with 50 μg mL^−1^ kanamycin and incubated at 37 °C in a shaker incubator overnight. The next day, 40 ml from the pre-culture was inoculated into each 1.5 L TB media with phosphate buffer containing 2.8 L Fernbach flasks supplemented with 50 μg mL^−1^ kanamycin (total 4.5 L culture) and cultures were incubated at 37 °C with 250 rpm shaking. When the cell density reached OD_600_ ∼1.0 to 1.1, culture flasks were placed in an ice-water bath for 45 min, then induced with 1 mM IPTG. At the point of induction, ferrous ammonium sulfate was also added into each flask (212 μM final concentration in each). Cultures were incubated at 16 °C with 200 rpm shaking overnight. Cells were harvested by centrifuging at 4 °C for 10 min. The wet cell pellet was flash-frozen in liquid nitrogen and stored at −80 °C until purification. Cell lysis and isolation of HisTag SdaA took place in a Coy anaerobic chamber. 45 g of frozen cell paste was lysed in 90 ml of lysis buffer: 50 mM HEPES pH 7.5, 100 mM KCl containing 8 mg lysozyme, ∼0.1 mg DNase, 1000 mg Triton X-100, 200 mg MgCl_2_ and two EDTA-free Pierce protease inhibitor tablets. After 30 min incubation at ambient temperature with continuous stirring, cell lysate was clarified by centrifuging at 17,000 rpm, 4 °C for 1 h. The resulting lysate supernatant was loaded to 20 ml HisPrep FF 16/10 (Cytiva) column equilibrated with 50 mM HEPES pH 7.5, 100 mM KCl, 10 mM imidazole *via* ÄKTA Start system. A step gradient was applied with increasing concentrations of imidazole (25 mM, 50 mM, 100 mM, 225 mM, and 500 mM) in the buffer. The resulting dark brown elution fraction was desalted through a desalting column packed with Sephadex G-25 resin to remove imidazole, and concentrated with Amicon Ultra-15 centrifugal filter (30 kDa MWCO). The iron content of the desalted and concentrated SdaA stock was determined to be 1.75 ± 0.04 Fe/SdaA monomer *via* atomic absorption (AA) spectroscopy. As-purified SdaA was chemically reconstituted in 50 mM HEPES pH 7.5, 100 mM KCl buffer supplemented with 0.6 mM ferric chloride (FeCl_3_), 0.6 mM sodium sulfide nonahydrate (Na_2_S·9H_2_O) and 5 mM dithiothreitol (DTT). The reconstitution mixture was incubated on a gel ice pack until the broad absorption peak of [4Fe-4S] at ∼410 nm stabilized (∼2.5 h). Then, the mixture was centrifuged at 13,000 rpm for 10 min and desalted through a desalting column packed with Sephadex G-25 resin to remove excess iron, sulfur, and Fe-S cluster polymers. The resulting stock of SdaA was concentrated with Amicon Ultra-15 Centrifugal Filter (30 kDa MWCO) and the iron content of the protein stock was analyzed with AA spectroscopy. Aliquoted protein stocks were flash-frozen with liquid nitrogen and stored at −80 °C.

### Serine deaminase (SdaA)-coupled enzyme activity assay

A previously published protocol was followed with some modifications (51). SdaA catalyzes the cleavage of the C_β¯_NH_2_ bond of serine to yield pyruvate and ammonium. To determine the activity of SdaA, the deamination reaction was coupled to the NADH-dependent reaction of lactate dehydrogenase (LDH, EC 1.1.1.27) which reduces pyruvate to lactate. L-serine, NADH, and lyophilized LDH were dissolved in 20 mM Tris-HCl pH 8.0 to prepare stock solutions. The final concentration of the reaction components is as follows: 5 mM L-serine, 0.3 mM NADH, 1 U LDH, and 7 nM SdaA in total 1 ml. L-serine, NADH, and LDH were premixed and transferred into a septum-sealed quartz cuvette in the glovebox. Then, the pre-mix was taken outside of the glovebox and placed into a Cary 60 UV-vis spectrometer equipped with RTE-5 refrigerated circulating bath set to 37 °C to equilibrate the pre-mix temperature prior to initiating the reaction by adding SdaA. 10 μl of SdaA was measured in the glovebox and transferred outside the glovebox in a gastight Hamilton syringe. The needle of the syringe was stabbed into an empty crimp vial sealed inside the glovebox. The reaction started by adding 10 μl of SdaA into the 37 °C premix through the septa and mixed quickly. UV-vis scans were recorded, and the reaction was monitored focusing on the decrease in the NADH absorption at 340 nm (ε_340nm_ = 6022 M^−1^ cm^−1^). The time-dependent decrease in the absorption of NADH indicates that SdaA cleaves serine and produces pyruvate, then LDH reduces pyruvate leading to oxidation of NADH to NAD^+^. Absorbance at each time point was used to calculate the amount of NADH remaining (μM) and plotted against time (min). The slope of the linear region was used as the rate of NADH consumption by LDH as a proxy to serine deamination by SdaA. The activity of SdaA towards the deamination of serine was found to be 130 μM serine min^−1^ which corresponds to a k_cat_ value of 303 s^−1^.

### E. coli aspartate ammonia-lyase (AspA) preparation

The *E. coli aspA* gene was purchased from Genscript. The DNA sequence was codon-optimized for expression in *E. coli* and was cloned into a pET-23a vector between the *Nhe*I and *EcoR*I restriction sites, allowing for expression of aspartate ammonia lyase with a C-terminal His_6_ tag in *E. coli* BL21(DE3) cells. The corresponding DNA sequence is provided in the Supporting Information.

Pre-cultures were started in 60 ml Luria-Bertani (LB) media supplemented with 100 μg mL^−1^ ampicillin and incubated at 37 °C overnight. The next day, 40 ml pre-culture was inoculated into each 1.5 L (total 4.5 L) potassium phosphate buffered Terrific-Broth media with 100 μg mL^−1^ ampicillin and incubated at 37 °C with 250 rpm shaking. When cell density reached OD_600_ ∼1.2 to 1.3, culture flasks were put into an ice-water bath for 45 min. After cold shock, cells were induced with 1 mM isopropyl ß-D-1-thiogalactopyranoside (IPTG) and incubated at 16 °C with 200 rpm shaking overnight. Cells were centrifuged at 4 °C for 10 min. The wet cell pellet was flash-frozen in liquid nitrogen and stored at −80 °C until further use.

To isolate his-tagged AspA, all lysis and purification steps were performed in a Coy anaerobic chamber. Lysis was performed at a ratio of 2 ml lysis buffer per 1 g of cell paste and ambient temperature in 50 mM Tris-HCl pH 7.5, and 100 mM KCl containing the same lysis buffer components outlined above for SdaA. After incubation cell lysate was clarified by centrifuging at 17,000 rpm for 1 h. Clarified lysate was loaded to the Ni-NTA column and equilibrated with 50 mM Tris-HCl pH 7.5, 100 mM KCl, and 10 mM imidazole buffer. Imidazole concentration is increased following a step gradient from 25 mM, 50 mM, 150 mM, 225 mM, and 500 mM. Fraction collected from the elution with 50 mM Tris-HCl pH 7.5, 100 mM KCl at 150 mM imidazole was loaded onto a HiPrep 26/10 desalting column to remove imidazole. Desalted AspA was concentrated with Amicon Ultra-15 centrifugal filter (30 kDa MWCO). The resulting protein was aliquoted and flash-frozen with liquid nitrogen and stored at −80 °C until further use.

### Aspartate ammonia-lyase (AspA) coupled activity assay

Aspartate ammonia-lyase catalyzes the deamination of aspartate to ammonia and fumarate. The ammonia production of AspA from L-aspartate was coupled to the NADH-dependent reductive amination of α-ketoglutarate by glutamate dehydrogenase (GDH from beef liver, EC 1.4.1.3). The reaction took place outside of the glovebox at ambient temperature (∼25 °C) in 1 M Tris-HCl pH 8.0 buffer. L-aspartate, α-ketoglutarate, NADH, ADP, and GDH were dissolved in a buffer. The reaction contained 10 mM L-aspartate, 5 mM MgCl_2_, 10 mM α-ketoglutarate, 0.3 mM NADH, 0.5 mM ADP, 10 U GDH, and 50 nM monomeric AspA. The reaction was initiated with the addition of AspA and the decrease in 340 nm absorption signal of NADH (ε_340nm_ = 6.22 mM^−1^ cm^−1^) was monitored. The concentration of NADH at each time point was determined and plotted against time. The rate of the NADH oxidation was calculated as 68 μM NADH min^−1^, which corresponds to a k_cat_ value of 22.6 s^−1^.

### SAM Synthesis

Preparation of enzymatically synthesized SAM was performed as described previously (72, 73, 74).

### In vitro [FeFe]-Hydrogenase maturation reactions

The *in vitro* maturation reactions were adapted from previously reported procedures (14) as follows. *In vitro* maturation of *Cr*HydA was carried out at an ambient temperature in an anaerobic MBraun chamber (O_2_ ≤ 1 ppm). Standard assays contained HydA (4 μM), HydE (5 μM), HydF (5 μM), HydG (25 μM), Fe(II) (6.4 mM), l-cysteine (2 mM), l-tyrosine (2 mM), l-serine (50 mM), NH_4_Cl (up to 50 mM), SAM (2.5 mM), GTP (20 mM), MgCl_2_ (20 mM), dithionite (2 mM), PLP (1 mM), DTT (1 mM), T-protein (10 μM), and SHMT (5 μM). For assays involving the amino acid ammonia lyases, NH_4_Cl was omitted and either AspA and aspartate or SdaA and serine was added, as detailed below. Pyruvate was added at the concentrations indicated for the experiments shown in Fig. S8. Assay components were incubated together (200 μl final volume) for 12 h in a 100 mM HEPES, pH 8.2, 50 mM KCl buffer, prior to removing an aliquot to assay for active hydrogenase.

When *in vitro* maturation of *Cr*HydA was carried out with AspA, the pH of the stock solution of aspartate (50 mM final concentration) was adjusted to 8 with a solution of KOH and added slowly over 2 h to AspA (23 nM final concentration) before adding the components of HydA maturation (to 20 mM final concentration of aspartate). When *in vitro* maturation of *Cr*HydA was carried out with SdaA, the latter enzyme (17 nM final concentration) was incubated with 2 mM serine for 2 h before adding the components of HydA maturation along with 20 mM serine.

### [FeFe]-hydrogenase activity assays

The [FeFe]-hydrogenase activity assays were carried out in 2 ml reaction mixtures containing 2 μl of the maturation reaction mixture and freshly prepared 20 mM DT and 10 mM methyl viologen in buffer containing 50 mM Tris and 10 mM KCl pH 6.9. After 3 min, headspace gas (100 μl) was removed from the sealed crimp vial with a Hamilton gas-tight syringe. H_2_ production was quantified using a SHIMADZU GC-2014 equipped with a TCD detector using N_2_ as a carrier gas.

### Re-purification of holo-CrHydA from in vitro maturation experiments

The repurification of strep-tagged holo-*Cr*HydA was carried out anaerobically and followed a previously published protocol (14).

### EPR and ENDOR sample preparation

Q-band EPR and ENDOR samples were prepared with freshly purified holo-HydA in an MBraun glovebox with 100% N_2_ atmosphere. Matured holo-HydA was mixed with 2 mM thionin acetate from a freshly prepared stock solution, then transferred into an EPR tube, capped with a rubber septum, and then immediately transferred from the chamber and flash frozen in liquid N_2_ within ≤1.5 min. Samples were stored in liquid N_2_ until spectral acquisition occurred.

### EPR and ENDOR spectroscopy

35 GHz pulse EPR/ENDOR spectra were collected on a spectrometer described previously (75, 76) that is equipped with a helium immersion dewar for measurement at 2K. For a single molecular orientation and for the ^15^N nuclear spin with I = half, the ENDOR transitions for the ms = ±half electron manifolds are observed, to first order, at frequencies, where ν_n_ is the nuclear Larmor frequency, and A is the orientation-dependent hyperfine coupling (Equation 1). In the Mims experiment, the ENDOR intensities are modulated by an inherent response factor (R), R ∼ [1 - cos(2πAτ)] where τ is the interval between the first and second pulses in the three-pulse Mims spin-echo sequence. When Aτ = n (n = 0, 1, 2…), the ENDOR response is at a minimum, resulting in hyperfine “suppression holes’’ in the Mims spectra. In Figure 3 these holes are indicated by (↓).

(1) I=12:ʋ±=|ʋn±A2|

### Generation of sequence similarity networks

Each of the HydE proteins from the organisms of interest was run through BLAST using the EBI’s UniProt (77) BLAST webservice (default settings, with an E-Threshold of 0.00001, the maximum number of results 250, and against the full UniProtKB, Release 2024_03. For each BLAST run, the proteins were further filtered to remove any hits below 1e-50 to minimize the chance of pulling in related proteins that are not HydE, as BioB, PylB, and HmdB are all functions that belong to the same subgroup within the radical SAM superfamily (48). This took us to an input list of 1524 proteins.

The list of proteins was combined and made unique, then uploaded to the EFI sequence similarity network tool (78, 79) (https://efi.igb.illinois.edu/efi-est/) using the Accession IDs tab. The SSN threshold was chosen as 1e-95 to minimize the number of edges whilst retaining the maximum connectivity information, and identical proteins (100% sequence identity) were also filtered out, resulting in 1349 nodes and 215,924 edges.

The *hydF* (1465 nodes and 798,235 edges at 1e-95) network was generated in the same manner.

### Building the genomic context networks

Once the SSN had been generated, it was fed into the EFI’s Genome Neighbourhood Tool (https://efi.igb.illinois.edu/efi-gnt/) (78, 79) using the SSN generated in step 1 as the input. The neighborhood size (number of ORFs) was chosen to be ± 10 or ± 20 and includes RNAs in the placement count, although RNA components are discarded from any further analysis by the tool. The versions of underlying databases used in this analysis as part of the EFI’s toolkit is UniProt 2024_01, and the ENA version downloaded in January 2024.

The original SSN was then annotated with the results of the GCN and analyzed using Cytoscape (80).

Each of the genes of interest was examined for conserved Pfam domains (see Fig. 5) and these domains were searched for in the SSN (one of the annotations in the GCN output is the list of the Pfam domains for all the proteins in ± 10 or ± 20 ORFs).

## Data availability

Data described in this manuscript has been deposited at https://osf.io/p4znk/and is publicly available.

## Supporting information

This article contains supporting information including experimental procedures and supporting data.

## Conflicts of interest

The authors declare that they have no conflicts of interest with the contents of this article.
