# Supplementary material for: Role of ammonia-lyases in the synthesis of the dithiomethylamine ligand during [FeFe]-hydrogenase maturation
Source: J Biol Chem. 2024 Sep 10;300(10):107760. doi: 10.1016/j.jbc.2024.107760 (PMC11736057; doi:10.1016/j.jbc.2024.107760)
Supplement: Supporting Information [file mmc1.pdf]

Supporting Information for

Role of Ammonia Lyases in the Synthesis of the Dithiomethylamine Ligand During [FeFe]-hydrogenase Maturation

Adrien Pagnier[a], Batuhan Balci[a], Eric M. Shepard[a], Hao Yang[b], Alex Drena[b], Gemma L. Holliday[c], Brian M. Hoffman[b], William E. Broderick[a], and Joan B. Broderick[a*]

From the [a]Department of Chemistry and Biochemistry, Montana State University, Bozeman, MT, 59717, USA, and the [b]Department of Chemistry, Northwestern University, Evanston, IL. 60208, USA. [c]Digitisation, Pharmaceutical Science, Biopharmaceuticals R&D, AstraZeneca, Macclesfield, United Kingdom

*Corresponding author. Email: jbroderick@montana.edu. Phone: 406-994-6160

This SI contains Supplemental Methods, Figures S1-S8

Supplemental Methods

***Escherichia coli* Aspartate Ammonia-Lyase (AspA) Optimized Sequence**
The corresponding codon-optimized DNA sequence for *E. coli* AspA is as follows:
atggctagcATGAGCAACAACATCCGTATTGAGGAAGACCTGCTGGGCACCCGTGAAGTTCCGGCGGAT
GCGTACTATGGTGTGCACACCCTGCGTGCGATCGAAAACTTCTACATTAGCAACAACAAGATCAGCG
ACATTCCGGAGTTTGTTCGTGGTATGGTGATGGTTAAGAAAGCGGCGGCGATGGCGAACAAGGAAC
TGCAGACCATCCCGAAAAGCGTTGCGAACGCGATCATTGCGGCGTGCGATGAGGTGCTGAACAACG
GCAAATGCATGGACCAGTTCCCGGTGGATGTTTACCAAGGTGGCGCGGGTACCAGCGTTAACATGA
ACACCAACGAGGTGCTGGCGAACATTGGCCTGGAACTGATGGGTCACCAGAAGGGCGAGTACCAAT
ATCTGAACCCGAACGACCACGTGAACAAATGCCAAAGCACCAACGATGCGTACCCGACCGGTTTTC
GTATCGCGGTTTATAGCAGCCTGATCAAACTGGTGGACGCGATTAACCAGCTGCGTGAGGGCTTCG
AGCGTAAGGCGGTTGAATTTCAAGACATTCTGAAAATGGGTCGTACCCAGCTGCAAGATGCGGTGC
CGATGACCCTGGGCCAGGAGTTCCGTGCGTTTAGCATCCTGCTGAAGGAAGAGGTGAAAAACATTC
AACGTACCGCGGAGCTGCTGCTGGAAGTGAACCTGGGTGCGACCGCGATTGGTACCGGTCTGAAC
ACCCCGAAGGAATACAGCCCGCTGGCGGTGAAGAAGCTGGCGGAAGTGACCGGTTTTCCGTGCGT
GCCGGCGGAGGACCTGATCGAAGCGACCAGCGATTGCGGCGCGTATGTGATGGTTCACGGTGCGC
TGAAACGTCTGGCGGTTAAGATGAGCAAAATTTGCAACGACCTGCGTCTGCTGAGCAGCGGTCCGC
GTGCGGGCCTGAACGAGATCAACCTGCCGGAACTGCAGGCGGGTAGCAGCATTATGCCGGCGAAG
GTTAACCCGGTGGTTCCGGAAGTGGTTAACCAAGTGTGCTTCAAAGTTATCGGCAACGATACCACCG
TGACCATGGCGGCGGAGGCGGGTCAGCTGCAACTGAACGTGATGGAACCGGTTATCGGCCAGGCG
ATGTTTGAGAGCGTTCACATTCTGACCAACGCGTGCTACAACCTGCTGGAAAAGTGCATCAACGGTA
TTACCGCGAACAAAGAGGTGTGCGAAGGCTACGTTTATAACAGCATCGGTATTGTGACCTATCTGAA
CCCGTTCATCGGTCACCACAACGGCGACATCGTTGGCAAGATTTGCGCGGAGACCGGCAAAAGCGT
GCGTGAGGTGGTTCTGGAACGTGGTCTGCTGACCGAGGCGGAACTGGACGATATTTTTAGCGTGCA
GAACCTGATGCACCCGGCGTACAAGGCGAAACGTTATACCGATGAGAGCGAACAAGCGAATTCGAG
CTCCGTCGACAAGCTTGCGGCCGCACTCGAGCACCACCACCACCACCAC

Which leads to the following protein sequence:
MASMSNNIRIEEDLLGTREVPADAYYGVHTLRAIENFYISNNKISDIPEFVRGMVMVKKAAAMANKELQTIP
KSVANAIIAACDEVLNNGKCMDQFPVDVYQGGAGTSVNMNTNEVLANIGLELMGHQKGEYQYLNPNDHV
NKCQSTNDAYPTGFRIAVYSSLIKLVDAINQLREGFERKAVEFQDILKMGRTQLQDAVPMTLGQEFRAFSI
LLKEEVKNIQRTAELLLEVNLGATAIGTGLNTPKEYSPLAVKKLAEVTGFPCVPAEDLIEATSDCGAYVMVH
GALKRLAVKMSKICNDLRLLSSGPRAGLNEINLPELQAGSSIMPAKVNPVVPEVVNQVCFKVIGNDTTVTM
AAEAGQLQLNVMEPVIGQAMFESVHILTNACYNLLEKCINGITANKEVCEGYVYNSIGIVTYLNPFIGHHNG
DIVGKICAETGKSVREVVLERGLLTEAELDDIFSVQNLMHPAYKAKRYTDESEQANSSSVDKLAAALEHHH
HHH

Bioinformatics analysis
Analysis of *hydE* genes revealed that the number of proteins in the SSN that have at least one of the other *hyd* maturase genes in the vicinity of *hydE* is 958 out of 1,349 (roughly 70 %); this is less than 100 % since using BLAST to expand on the protein space will inevitably pull back pseudo-HydE proteins, proteins that look very similar to HydE but don't have the HydE function. The total number of nodes with at least one of the *aspA*, *ilvA*, or *sdaA* genes (as determined by the Pfam domain membership) is 408, or roughly 42 % of the authentic *hydE* genes, i.e. those which are also adjacent to at least one other *hyd* gene (**Figure S3**).

Supplemental Figures

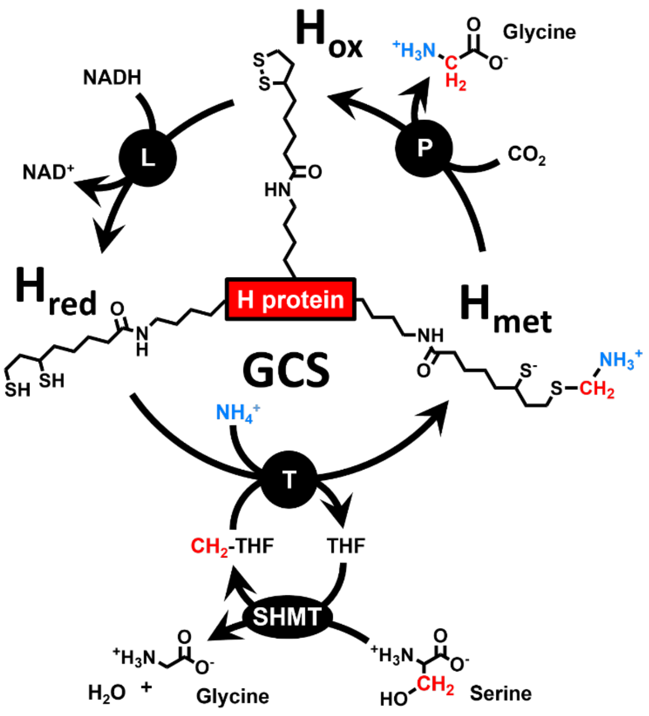

Figure S1. Schematic representation of the glycine cleavage system.

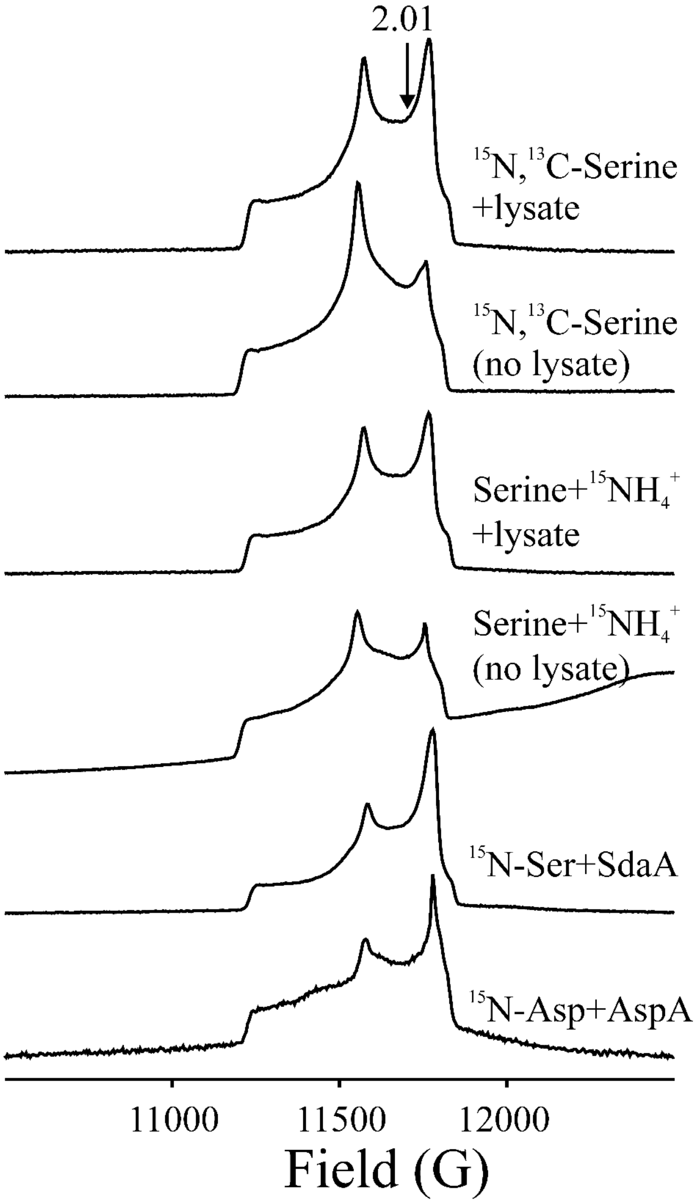

Figure S2. Q-band pulse EPR spectra of the matured HydA after re-purification from the maturation mixtures, the arrow indicates the field g = 2.01 where ENDOR is interrogated, T = 2K.

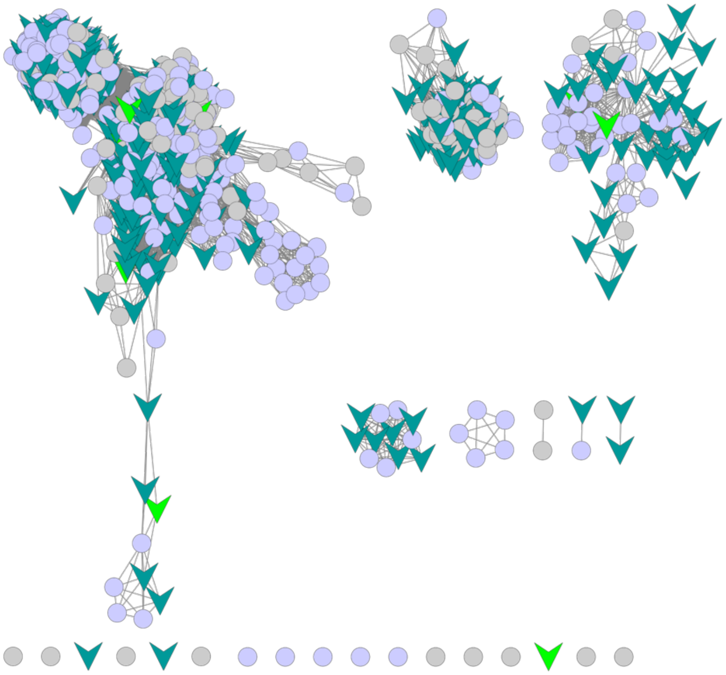

HydE					
Gene	Pfam Domains			+/- 20	
				# Nodes	%
hydG	PF04055	PF06968		856	63.45
hydF	PF18128	PF18133	PF01926	786	58.27
aspA	PF10415	PF00206		219	16.23
ilvA	PF00585	PF00291	PF01842	193	14.31
sdaA	PF03315	PF03313	PF01842	41	3.04
**Total Number Nodes**				**1349**	
**Total *aspA*, *ilvA*, and *sdaA***				408	30.24
**Total *hyd***				958	71.02

Figure S3. Top, Sequence similarity network showing the HydE protein set, the original query proteins are shown as a light green "V" shape, the proteins with at least one of the Pfam domains of interest are shown as teal "V's" and those proteins that have at least one of the hyd gene Pfam domains, but no other Pfam domain of interest are shown as pale purple circles. Figure created using Cytoscape and a perfuse force directed layout algorithm. Bottom, genes of interest and their associated Pfam domain, the number of nodes for which at least one of those Pfam domains was identified as a genomic neighbour of *hydE* within the +/- 20 ORF, and the percentage of the total number of nodes represented

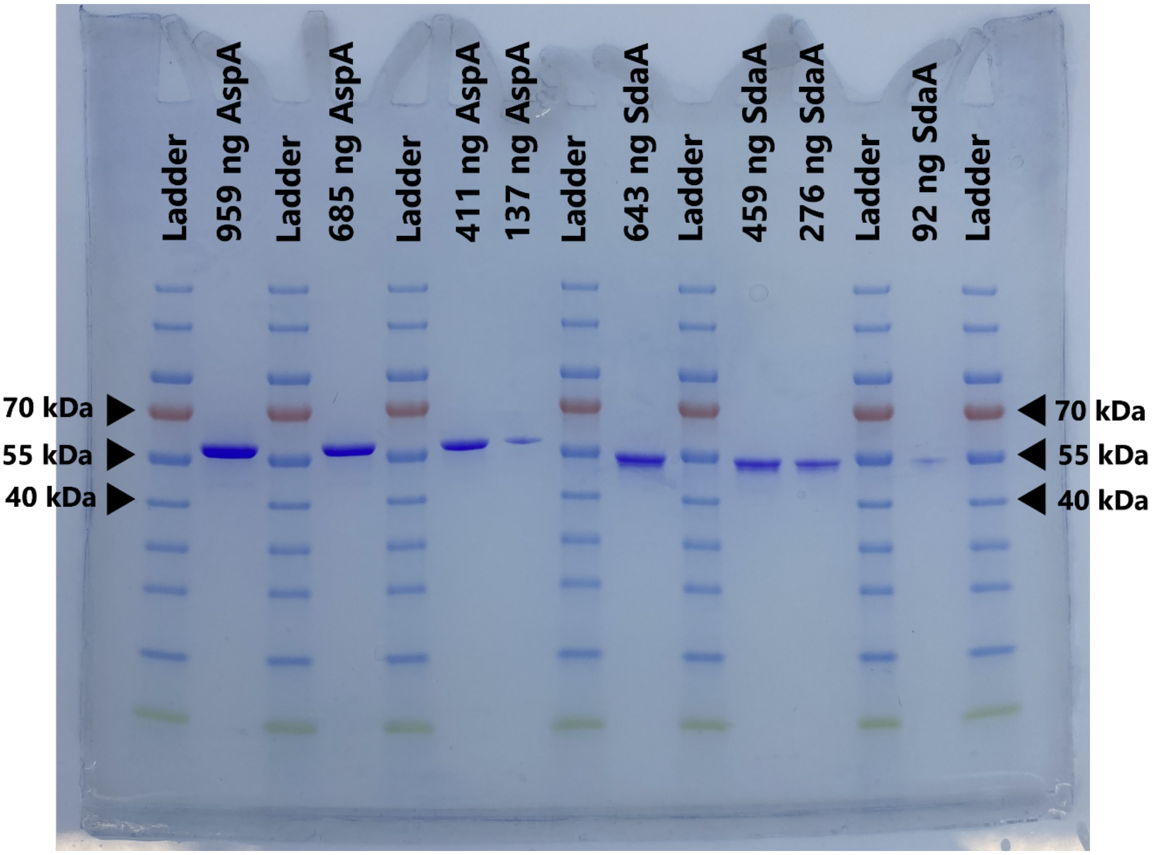

Figure S4. SDS-PAGE gel of purified AspA and SdaA proteins used in the maturation assays of HydA. Theoretical molecular weights are AspA: 54.8 kDa and SdaA: 51.1 kDa.

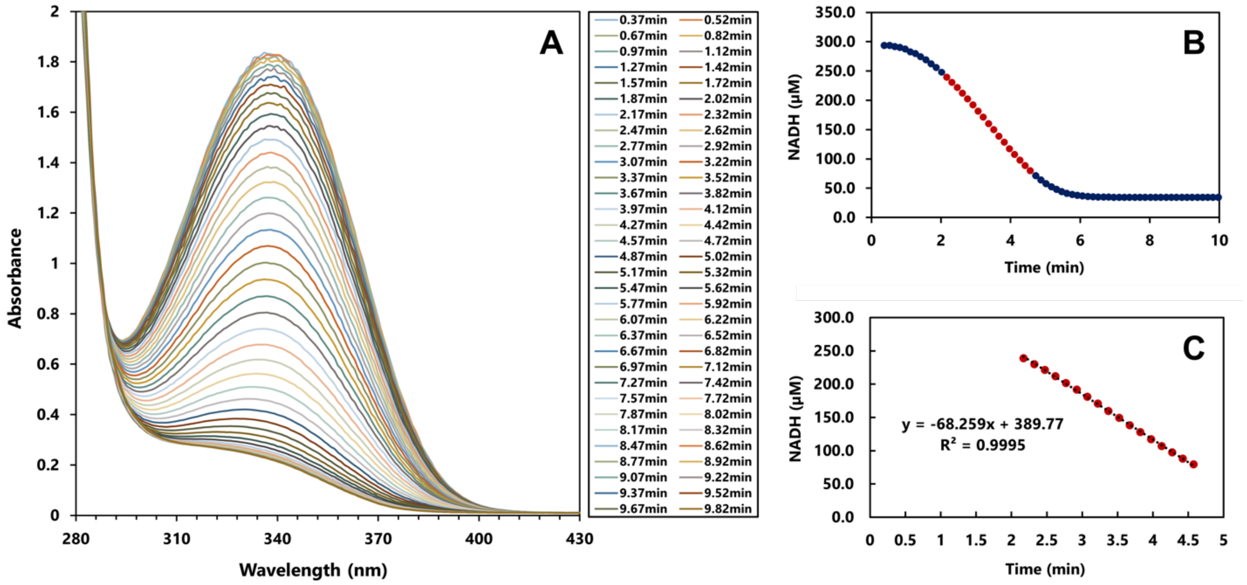

Figure S5. The GDH-coupled AspA activity assay results. **(A)** UV-vis scans of the coupled reaction focusing on the 340 nm region which indicates time-dependent decrease of NADH in the reaction. **(B)** Calculated concentration (μM) of NADH plotted against time. The linear region indicated with red dots. **(C)** The linear region from the panel B. The slope of the trendline indicated the reaction rate of NADH oxidation therefore the deamination of aspartate by AspA.

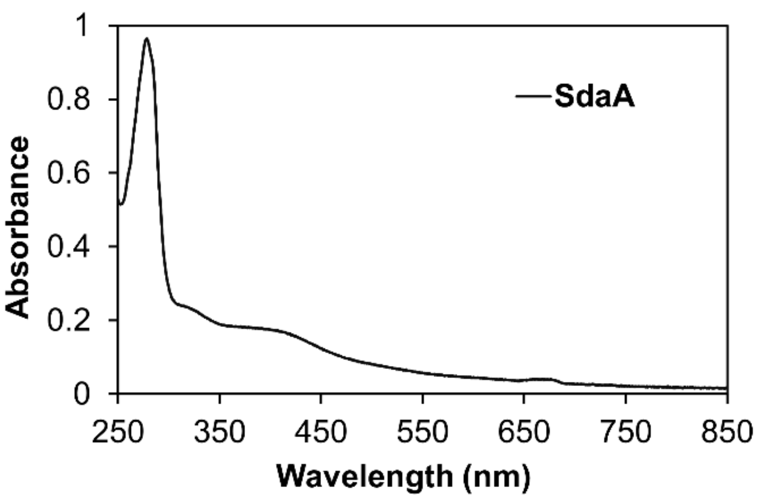

Figure S6. UV-visible absorption spectrum of reconstituted and desalted SdaA diluted in 50 mM HEPES pH 7.5, 100 mM KCl to 9 μM with 4.0±0.1 Fe per SdaA monomer.

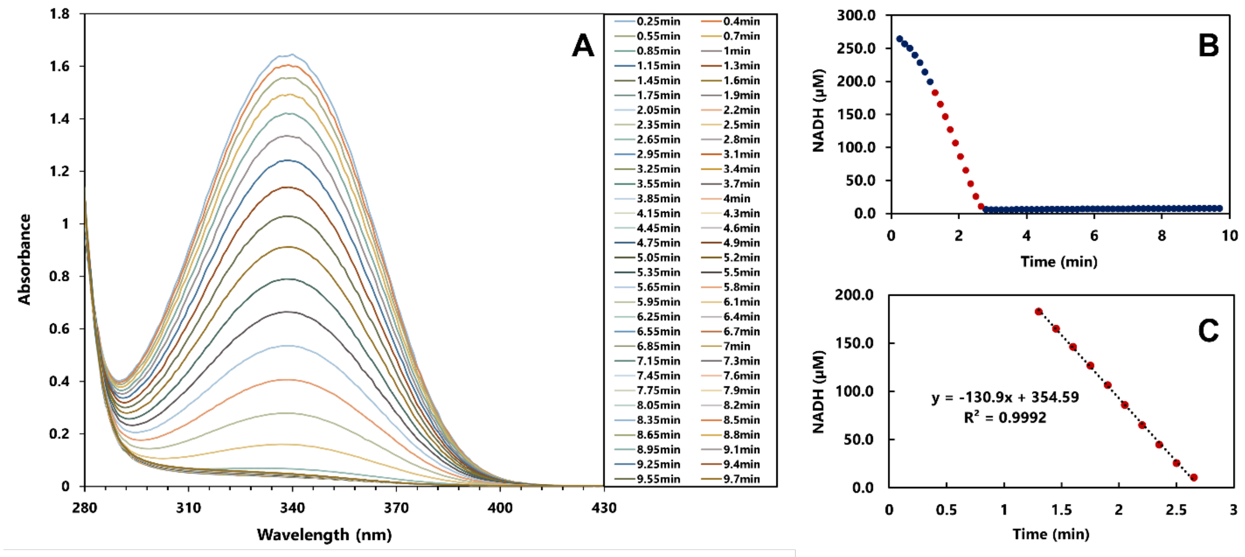

Figure S7. LDH-coupled SdaA activity assay results. **(A)** UV-vis scans from the continuous coupled reaction indicating the time-dependent decrease at 340 nm absorbance peak of NADH. **(B)** Demonstration of the reaction progress with calculated concentrations of NADH at each time point. Linear region indicated with red dots. **(C)** The linear region from the panel B. The slope from the trendline provided the reaction rate of NADH oxidation so that the deamination of serine.

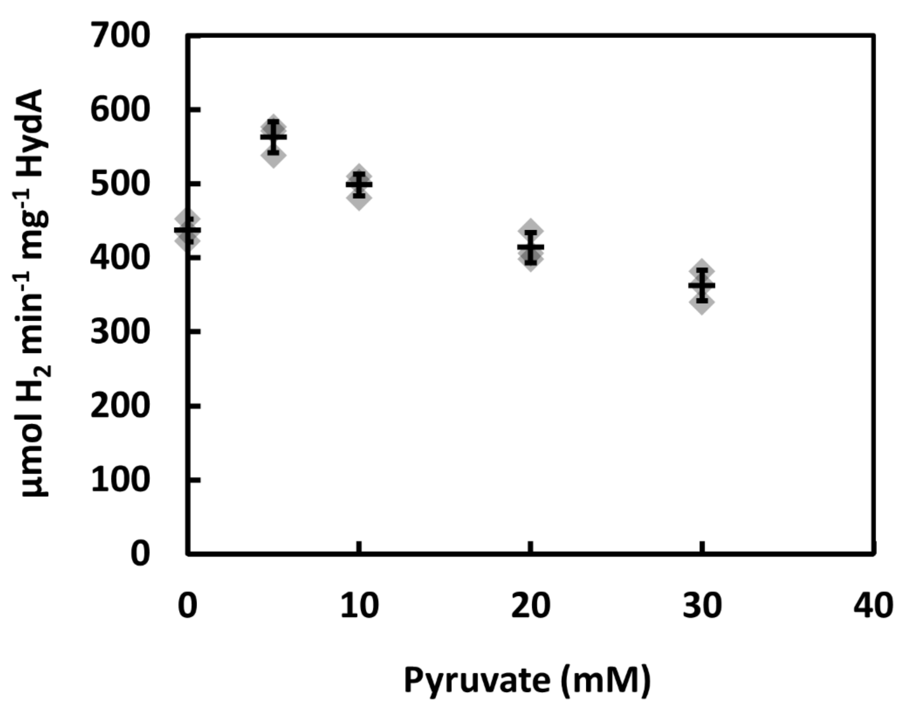

Figure S8. Impact of added pyruvate in [FeFe]-hydrogenase maturation reactions. Maturation reactions were carried out as described in the Experimental Methods section, with pyruvate added to the concentrations shown. Hydrogen production assays were carried out in triplicate, with individual values represented as diamonds at each concentration, with the mean and standard deviations overlaid on the data. Assay conditions are as described in the experimental methods section.
